# Endoscopic submucosal dissection combined with endoscopic hand-suturing for a superficial esophageal cancer in a diverticulum

**DOI:** 10.1055/a-2381-4993

**Published:** 2024-09-06

**Authors:** Shibo Song, Chen Zhang, Guiqi Wang, Lizhou Dou

**Affiliations:** 1 Endoscopy Center, Peking University First Hospital, Beijing, China; 2 26447 Department of Endoscopy, National Cancer Center/National Clinical Research Center for Cancer/Cancer Hospital, Chinese Academy of Medical Sciences and Peking Union Medical College, Beijing, China

Superficial esophageal cancer (SEC) occurring in a diverticulum is extremely rare 1 2 . Endoscopic submucosal dissection (ESD) is the preferred treatment for SEC, but diverticulum increases the risk of perforation, necessitating preventive measures. For the first time, we present a case in which an SEC in a diverticulum was safely and successfully cured using ESD combined with endoscopic hand-suturing (EHS).

A 65-year-old man was diagnosed with an SEC (28–31 cm from the incisors) during follow-up after radical gastrectomy ( Fig. 1 ). Despite the biopsy showing high grade intraepithelial neoplasia (HGIN), the patient underwent ESD due to its malignant potential.

**Fig. 1:**
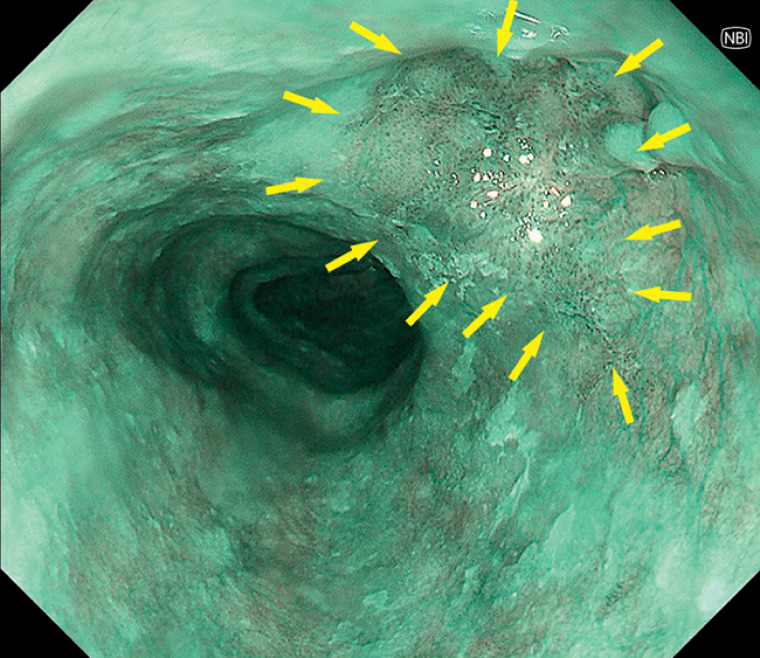
A superficial esophageal cancer (yellow arrows) on the surface of a diverticulum.

Labeling, submucosal injection, and submucosal dissection were performed according to ESD protocols. The lesion was removed en bloc without perforation ( Video 1 ). A depression of the muscularis propria was observed in the defect ( Fig. 2 ). To prevent delayed perforation, we sutured the depression using EHS ( Fig. 3 , Video 1 ). The prototype needle holder (entrusted manufacturer: Vedkang, Jiangsu, China) used for EHS was designed by our team ( Fig. 4 ). Notably, to achieve successful suturing, we made some modifications to the V-loc 180 needle with absorbable barbed suture (VLOCL0803; Covidien, Mansfield, Massachusetts, USA), including appropriately straightening the curvature of the needle and shortening the suture thread ( Fig. 5 ).

A superficial esophageal cancer on the surface of a diverticulum was completely removed via endoscopic submucosal dissection, and the depression of the muscularis propria in the defect was closed via endoscopic hand-suturing.Video 1

**Fig. 2:**
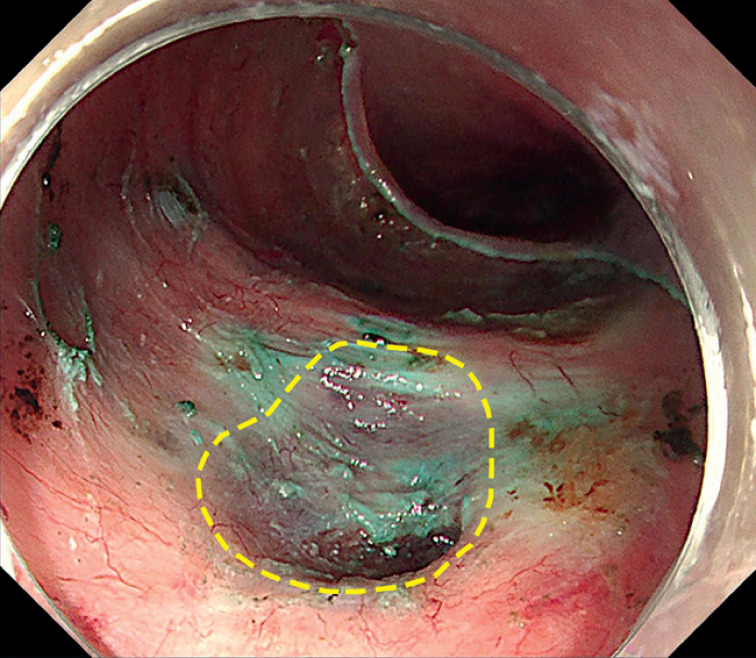
A depression of the muscularis propria (yellow circle) in the defect after endoscopic submucosal dissection.

**Fig. 3:**
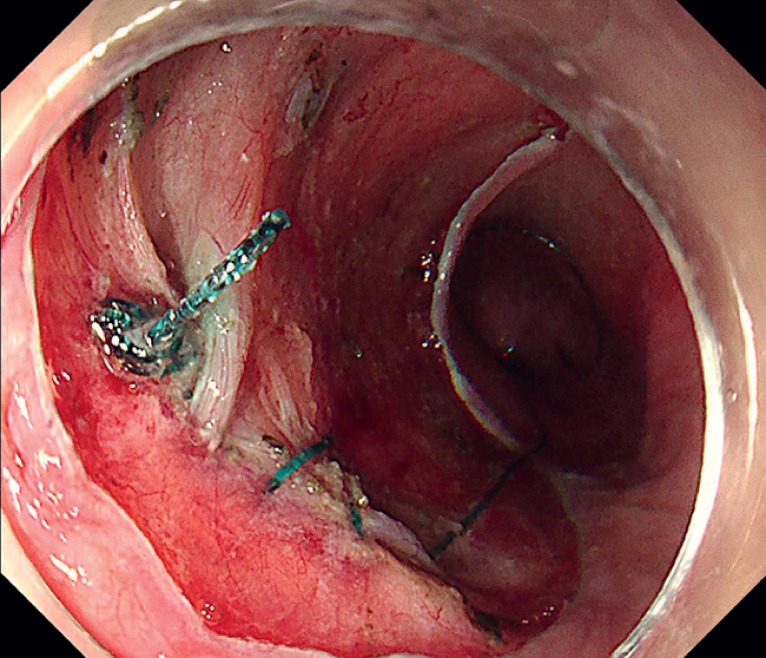
The closed depression in the defect.

**Fig. 4:**
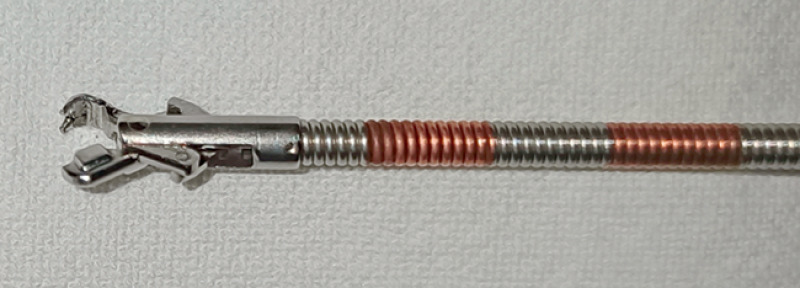
The prototype needle holder (entrusted manufacturer: Vedkang, Jiangsu, China) designed by our team.

**Fig. 5:**
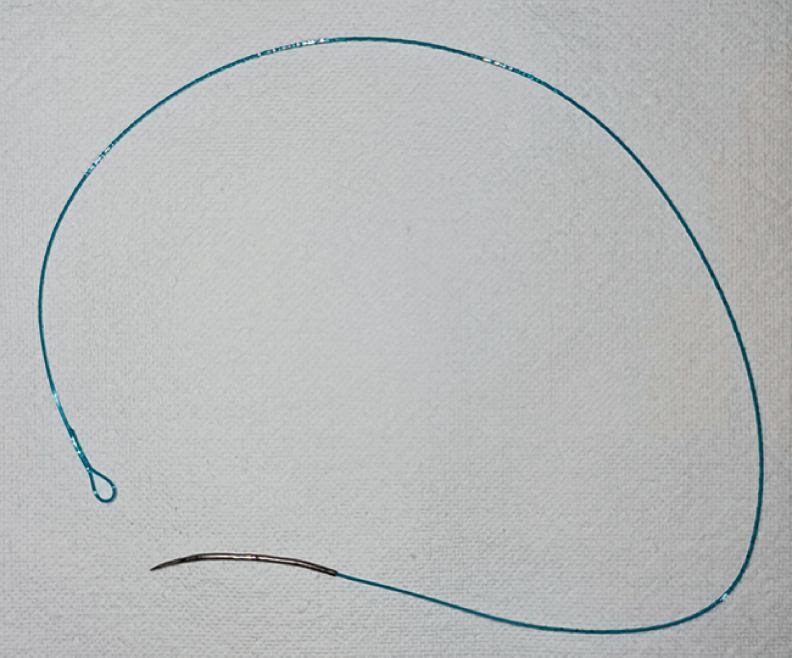
The modified V-loc 180 needle. The curvature of the needle was appropriately straightened and the suture thread was properly shortened.

The resection time and suture time were 31 minutes and 20 minutes, respectively. The patient was allowed to have a liquid diet on postoperative day 4 and was discharged on postoperative day 5 without adverse events. Histology confirmed complete resection of HGIN. The gastroscopy after 3 months showed good healing of the defect.

The use of ESD combined with EHS for treating SEC in a diverticulum has not been reported previously. In this case, the lesion was completely removed via ESD, and postoperative adverse events were effectively avoided via EHS. Further accumulation of clinical experience is desirable.

Endoscopy_UCTN_Code_TTT_1AO_2AG_3AD

## Notes

Endoscopy E-Videos https://eref.thieme.de/e-videos
*E-Videos* is an open access online section of the journal *Endoscopy* , reporting on interesting cases and new techniques in gastroenterological endoscopy. All papers include a high-quality video and are published with a Creative Commons CC-BY license. Endoscopy E-Videos qualify for HINARI discounts and waivers and eligibility is automatically checked during the submission process. We grant 100% waivers to articles whose corresponding authors are based in Group A countries and 50% waivers to those who are based in Group B countries as classified by Research4Life (see: https://www.research4life.org/access/eligibility/ ). This section has its own submission website at https://mc.manuscriptcentral.com/e-videos .
